# Fecal metabarcoding of the endangered Pacific pocket mouse (*Perognathus longimembris pacificus*) reveals a diverse and forb rich diet that reflects local habitat availability

**DOI:** 10.1002/ece3.10460

**Published:** 2023-09-21

**Authors:** Amy G. Vandergast, Cheryl S. Brehme, Deborah Iwanowicz, Robert S. Cornman, Devin Adsit‐Morris, Robert N. Fisher

**Affiliations:** ^1^ U.S. Geological Survey Western Ecological Research Center San Diego California USA; ^2^ U.S. Geological Survey, Eastern Ecological Science Center, Leetown Research Laboratory Kearneysville West Virginia USA; ^3^ U.S. Geological Survey Fort Collins Science Center Fort Collins Colorado USA

**Keywords:** amplicon sequencing, diet preference, fecal sampling, habitat restoration, Heteromyidae, ITS2 gene, noninvasive

## Abstract

Information on diet breadth and preference can assist in understanding links between food resources and population growth and inform habitat restoration for rare herbivores. We assessed the diet of the endangered Pacific pocket mouse using metabarcoding of fecal samples and compared it to plant community composition in long‐term study plots in two populations on Marine Corps Base Camp Pendleton, San Diego County, CA. Fecal samples (*n* = 221) were collected between spring 2016 and fall 2017 during monthly live‐trap surveys. Concurrently, percent cover and plant phenology were measured in plots centered on trap locations. Fecal samples were sequenced with paired‐end reads of the internal transcribed spacer 2 region of the nuclear ribosomal gene, and the resulting amplicons were matched to a regionally specific database. Seventy‐three plant taxa were detected, which were mostly forbs and perennial herbs (70–90%). Diet composition differed between populations, years, seasons, and plots. Overall, diet and local habitat composition in plots were significantly correlated. However, we detected some differences in above‐ground seed availability and proportion in fecal samples that indicate diet preferences for some forbs, perennial herbs, and native bunch grasses over perennial shrubs and non‐native grasses. This is the first study of PPM to pair plant phenology surveys with diet metabarcoding to estimate resource selection, and results suggest that managing habitat for diverse native forb communities and reducing non‐native grass cover may be beneficial for this critically endangered species.

## Graphical abstract

Fecal metabarcoding revealed high plant diversity, spatial and temporal differences in diet composition correlated with habitat availability, and a suggested preference for forbs and annual herbs in the Pacific pocket mouse in southern California. Results can help inform seed palettes for habitat restoration and reestablishment efforts for this critically endangered species. This work is highly relevant to resource managers and agencies tasked with protection and restoration of habitat for the Pacific pocket mouse. More broadly, it provides a methodological example applicable to herbivore conservation.

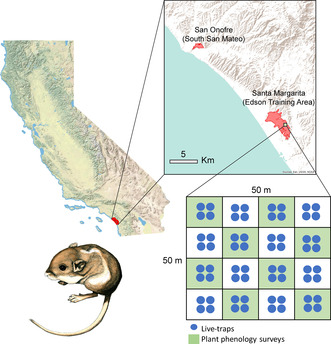

## 1 INTRODUCTION

Diet and resource availability can strongly influence survivorship, reproduction, and population abundance of mammals (White, 2008; Yunger, 2002), and food supply is a universal “bottom‐up” factor controlling population growth (e.g., Flowerdew et al., 2017; Ostfeld & Keesing, 2000; Sinclair & Krebs, 2002). In the case of conservation management of endangered mammals, delineating diet breadth and preference can provide critical guidance for restoration of degraded habitats to ensure that resources are available that can contribute to population persistence or growth (Ando et al., 2013; Kempel et al., 2015). Food quality and availability may be particularly important for small herbivorous and granivorous mammals such as heteromyid rodents, where food limitations are linked to increased torpor and weight loss in individuals (Tucker, 1966; Wolff & Bateman, 1978) and ultimately limit populations through declines in reproduction, survivorship, density, and abundance (Klinger, 2007; Predavec, 1994).

**FIGURE 1:**
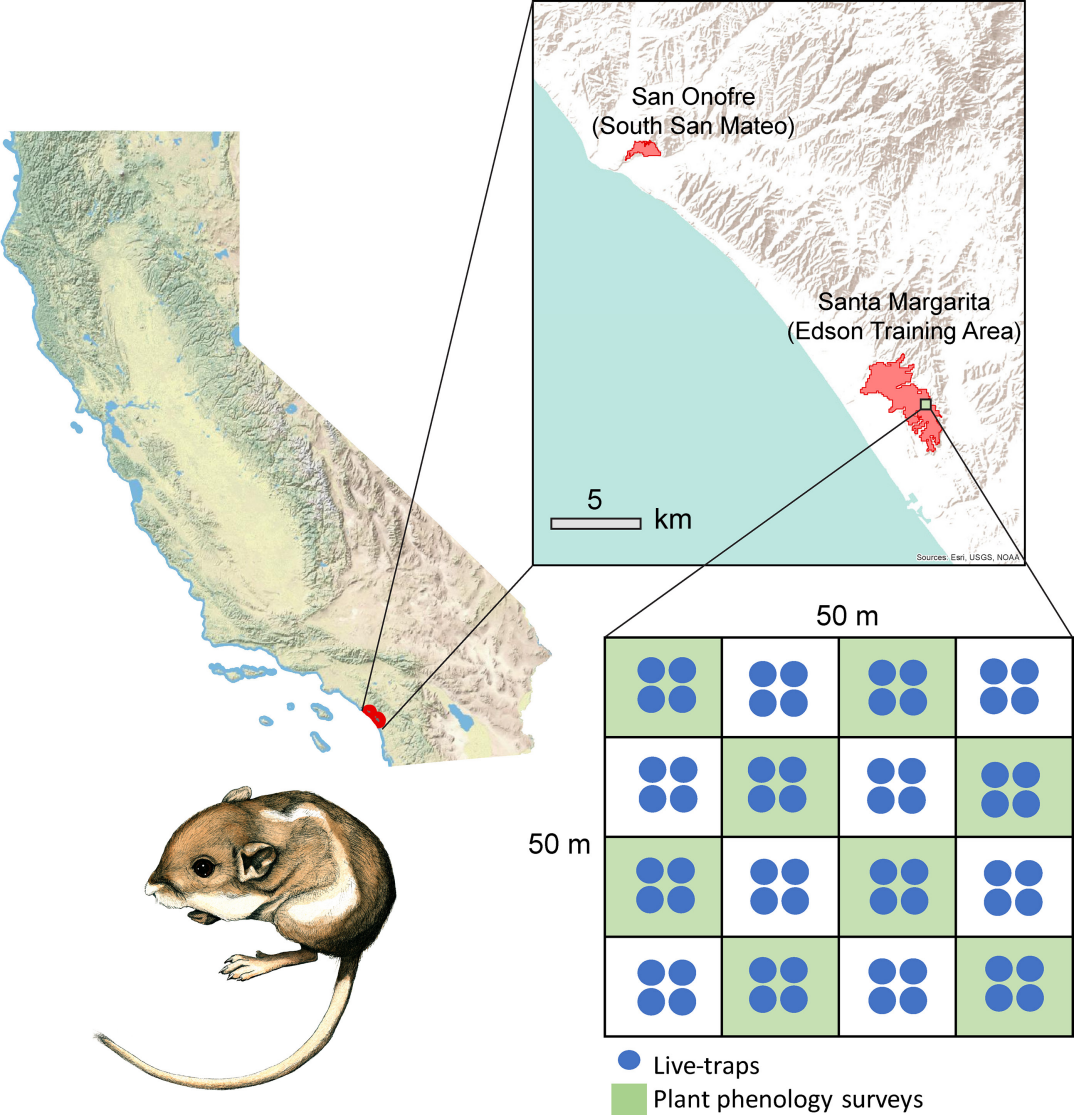
Map of study locations in southern California and layout of a 0.25 ha trapping grid with live traps shown in blue and vegetation cover and phenology survey areas shown in green). Pacific pocket mouse artwork (lower left) by Tristan Edgarian (USGS).

The Pacific pocket mouse (*Perognathus longimembris pacificus*; hereafter PPM) is a federally endangered member of the Heteromyidae family and one of the smallest rodents found in North America (USFWS, 1994; Williams et al., 1973). There are three extant wild populations of PPM (USFWS, 2020); two of these three are located within Marine Corps Base Camp Pendleton, San Diego County, CA (Figure 1). PPM is primarily found in open habitat with coastal scrub, grasses and forbs, and loose sandy soils (USFWS, 2020). Like other heteromyids, food availability appears to be a limiting factor linked to PPM activity patterns, behavior, and reproduction (Bartholomew & Cade, 1957; Brehme et al., In Review; Meserve, 1976). For example, PPM enter facultative torpor during the winter and in response to low resource conditions, and their above‐ground active season coincides with high annual forb availability (Brehme et al., In Review; Shier, 2008). Reproduction also fluctuates in response to available resources; there may be no reproduction in low‐resource years (Brehme et al., 2020). As is typical of heteromyids and indicative of food limitation, PPM collect and cache seeds below ground and within burrows for sustenance throughout the year.

Despite the apparent importance of food resources to population stability and persistence, prior to our fecal metabarcoding studies, direct studies of PPM diet in wild populations were limited to observations of cheek pouch contents (e.g., von Bloeker, 1931) and a single fecal microscopy study of *P. longimembris* from outside of the extant range of PPM (Meserve, 1976). Although these efforts identified the presence of some grasses and forbs, our initial metabarcoding analysis of PPM scat identified a much wider diet breadth of forbs and greater specificity than previously observed, and suggested that fecal metabarcoding could become a reliable non‐invasive method for further PPM diet characterization and resource selection studies in wild populations (Iwanowicz et al., 2016).

Here, we expand on previous work by pairing metabarcoding analysis of a large set of fecal samples from live‐trapped individuals with new site‐specific plant phenology surveys across a 2‐year period. Our goals were to document spatial and seasonal variation and preference in PPM diet in the two populations on MCBCP. Given the broad and variable diet characterized in Iwanowicz et al. (2016), we hypothesized that the diet would reflect seasonally available plant resources. Understanding the natural diet breadth and preferences of PPM could provide seed palettes for habitat restoration efforts for existing populations and inform checklists for receiver site selection efforts aimed at establishing new populations (Chock et al., 2022). Maintaining the three existing populations of PPM and establishing new populations are integral components of ongoing recovery efforts for this species (Chock et al., 2022; USFWS, 2020).

## 2 METHODS

### 2.1 Field collections

PPM fecal pellets were collected directly from live‐captured animals or collected from 9‐inch Sherman live‐traps in three 0.25‐ha trapping grids at San Onofre (South San Mateo site; hereafter SSM) and two 0.25‐ha grids at Santa Margarita (Edson Training Area site; hereafter EDSON) between March and July of 2016 and 2017 (Figure 1). Each trapping grid consisted of 64 traps placed among 16 0.016‐ha plots, with four traps subjectively placed within a 2 m diameter of the center of each plot. All Sherman traps were baited with sterilized millet seed (*Panicum miliaceum*). Live trapping events were conducted over three nights, where animals were assessed for age, sex, and reproductive condition. All PPM were permanently marked using VIE (visible implant elastomer) tags in the base of the tail (Shier, 2008). Each individual was given a unique 3‐digit number based on the combination of unique color and VID placement. While live‐trapping, fecal pellets were collected directly from individuals into a sterile microfuge tube during handling, and/or the trap was emptied into a bag, collecting scat and remaining bait seed. Fecal pellets were then sorted from bait seed in the laboratory using sterilized tweezers. In the field, traps were cleaned with sanitary wipes between all trap events to ensure there was no cross‐contamination of scat from other PPM individuals or other rodent species (Brehme et al., 2019). All trapping was conducted in accordance with FWS permit TE‐045994‐14 and California Scientific Collecting permits SCP‐838, SCP‐4186, SCP‐6488, and SCP‐7850. Fecal pellets were collected in envelopes with desiccant beads for rapid drying and stored at room temperature prior to extraction. Rapid drying on silica is a common collection technique known to result in high quality and quantity of DNA from plant tissues (Chase & Hills, 1991; Funk et al., 2017). This method has been applied in a number of fecal metabarcoding studies, many of which targeted plant‐based diets (Ando et al., 2020).

To estimate available seed resources in the habitat where PPM fecal pellets were collected, we conducted vegetation cover and plant phenology surveys at a subset of eight 0.016‐ha plots (every other plot) within each of the trapping grids concurrently with each live‐trap survey event (Figure 1). Trapping sessions and plant surveys were conducted every 4–6 weeks from April to September/October. In each trapping session, a single observer estimated vegetation cover and plant phenology on all survey visits each year to minimize observer bias and maintain consistency. Plant phenology surveys consisted of standardized visual vegetation estimates of cover type, including trees, shrubs, forbs, native grasses, non‐native grasses, and open ground. Small perennial herbs were included in the general forb category. Each live trapping location had a pin flag marking the center of a 12.5 × 12.5‐m grid, and each visual estimate of vegetation cover in the grid was performed within a 6‐m radius from the central location of the observer. A series of computer‐generated percent cover reference cards were used to increase the accuracy of visual estimates. For each plot, the total percent cover was equal to at least 100% but could be >100% if a cover type was overlapped with another cover type; for example, grass under a shrub would increase the cover at that plot. Next, each plant in that plot was identified to species or genus, and a visual estimate of percent cover was made for each taxon on the plot. Lastly, current phenology (one or more: new growth, budding, flowering, set seed, seeded, dead) was characterized for each species recorded on the plot. To estimate seed availability for each taxon, we summed the proportions of the taxon flowering, seeding, and newly dead (e.g., alive during the previous trapping session). We used this combined category to avoid underrepresenting available seed (it was noted in the field that some plants went from flowering to dead from one session to the next). Additionally, preliminary analyses of similarity suggested total proportions of flowering, seeding, and dead plants better fit the diet proportion data than seeding alone or flowering and seeding.

### 2.2 Amplification and library preparation

DNA extractions were performed at the Western Ecological Research Center San Diego Field Station laboratory. Dried fecal pellets were homogenized with a sterile pestle in a 1.5‐mL centrifuge tube and then extracted with the Qiagen DNeasy plant kit (Valencia, CA), which removes plant inhibitory compounds, following the manufacturer's protocols. Amplifications and sequencing were performed at the USGS Leetown Science Center Laboratory. Library preparation and sequencing generally followed methods in Iwanowicz et al. (2016) but used different primers that targeted the shorter ~350 bp internal transcribed spacer region 2 (ITS2). ITS2 is one of several plant barcoding genes commonly used in diet metabarcoding studies (Ando et al., 2020) and allowed us to utilize a comprehensive database of plant taxa collected locally in San Diego County: Bold Project SDH “*Barcoding the San Diego Natural History Museum* *Herbarium*” (Iwanowicz et al., 2016; Warne et al., 2015). In order to optimize read filtering and mapping parameters, we also prepared and sequenced two mock community samples consisting of known ratios of extracts of 23 plant species collected from PPM habitat. The mocks were identical in species composition to those used previously (Iwanowicz et al., 2016).

In all samples, ITS2 was initially amplified with standard primers (ITS2F: Chen et al., 2010; ITS4R: White et al., 1990; also see Sickel et al., 2015) at a 1 μM concentration. Polymerase chain reaction (PCR) cycling conditions consisted of an initial denaturation step of 95°C for 5 min, followed by 40 cycles of 30 s at 95°C, 35 s at 47°C, and 1.5 min at 72°C, and a final extension of 72°C for 10 min. Polymerase chain reaction (PCR) products of the appropriate size were confirmed with gel electrophoresis, cleaned with the Qiagen PCR Purification Kit (Valencia, CA), and quantified using the Qubit dsDNA HS Assay Kit (ThermoFisher Scientific). Samples were diluted in 10 mM Tris buffer (pH 8.5) to 5 ng/μL. Next, a library was created following the manufacturer's protocol from Illumina's 16S Metagenomic Sequencing Library preparation guide (Part #: 15,044,223 Rev. B). Libraries were quantified with the Qubit dsDNA HS Assay Kit, and DNA size spectra were determined using an Agilent DNA 1000 kit. Up to 96 samples were pooled and diluted to 4 nM using 10 mM Tris pH 8.5. A final 10 pM preparation was created with a 15% PhiX control spike following the manufacturer's protocol. DNA libraries were loaded onto an Illumina MiSeq with a 600‐v3 cartridge. Machine‐processed sequencing output has been deposited in the NCBI Sequence Read Archive under BioProject PRJNA801232.

### 2.3 Read filtering and mapping

**FIGURE A1:**
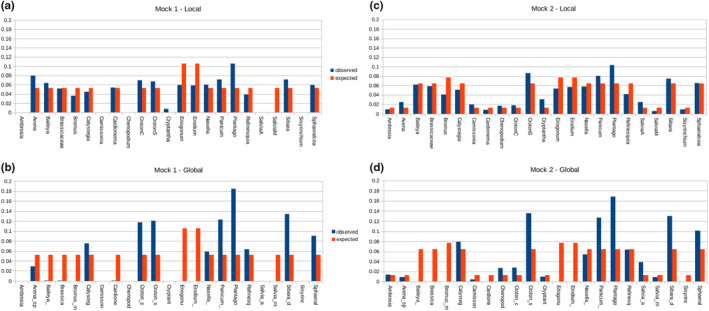
Mock libraries 1 (a, b) and 2 (c, d) sequenced for ITS2. Libraries were better recovered using a local alignment mode and a base score of 80 (a, c), rather than a global alignment mode (b, d). After local alignment, overall observed and expected proportions did not differ significantly.

Read processing of demultiplexed read pairs was performed with the bbmap package in the BBTools suite (Bushnell, 2016). Merged reads were trimmed with the bbduk script using *k* = 15 and mink = 11 to match exogenous sequences (primers and adapters) and q10 to trim poor quality bases at the 3′ end of reads prior to merging. The bbmerge script was implemented to merge read pairs using settings loose = t, *k* = 31. For failed merges, we applied additional quality trimming using qtrim2 = t and trimq = 15, after which the merge was repeated. Merged reads 200–450 bp in length were retained. Merged reads were mapped directly to the SDNHM regional ITS2 database (described above) with bowtie2 (Langmead & Salzberg, 2012). The very sensitive local alignment parameters were used with scoremin increased over the default to G,80,8 based on the results of Iwanowicz et al. (2016). The stringency of alignments was evaluated by parsing the CIGAR and MDZ strings of the SAM specification (Li et al., 2009). Alignments were kept if at least 98% were identical over aligned length, with a maximum of three gap positions. Skips of up to five positions were allowed at ends, which were counted as gaps of size 1. Thus, an alignment with allowable skips at both ends could have at most one additional gap position in the aligned portion of the read. These parameters provided good recovery of two mock communities prepared from extracted plant genomic DNA in known ratios and were much superior to global alignment only (Figure A1).

### 2.4 Taxonomic analysis

Counts of reads assigned to taxa were normalized as counts per million mapped reads. These were aggregated to the genus‐level, and genera with <1% of total reads in any individual sample were dropped from further analysis after first removing the genus *Panicum*, which is mostly comprised of millet seed used as bait (*P. miliaceum*). *Panicum* was present in 25% of all fecal samples and accounted for 9.9% of mapped reads. We chose to aggregate to genus‐level because previous simulation results suggested greater potential for mapping error among more closely related sequences (Iwanowicz et al., 2016), which are most likely found among conspecifics. Additionally, this level best matched the specificity of plant phenology field surveys. One exception was within the forb genus *Croton*. Field surveys detected the California native *Croton californicus* at SSM, which has previously been noted in high proportions in PPM diet (Iwanowicz et al., 2016), but only the more widely found *Croton setiger* occurred at EDSON; therefore, we separated these two species in analyses. Although imposing a 1% minimum threshold may remove rare components of the diet composition and thus underestimate richness, other studies have found it can dramatically reduce noise from contaminants (Ando et al., 2018). To further reduce possible sequence‐based misidentifications, we filtered taxa based on previous field survey results. Taxa were retained if they were recorded in any plant phenology surveys conducted in PPM trapping grids (described above) or, more broadly, in long‐term botanical surveys conducted by the San Diego Natural History Museum (SDNHM). SDNHM surveys were conducted between 2006 and 2019 adjacent to PPM sites (<2 km from PPM population boundaries, Plant Atlas Grid Squares C1‐2, F4‐5; http://sdplantatlas.org/, accessed February 2021). SDNHM surveys consisted of visual searches in which all observed plant species were recorded and new records were vouchered (M. Mulligan, personal communication).

### 2.5 Diet diversity and phenology

Using this filtered dataset, we calculated and compared species richness and community differences among populations, survey years, and seasons. Given that starting proportions of mock communities were well‐recovered (Figure A1), diet diversity and composition comparisons were made using the relative read abundance (RRA; proportions of total mapped reads; e.g., Deagle et al., 2019), although the frequency of observations in samples (FOO) were used for richness estimates and are also presented in Table A1. All multivariate analyses were conducted in Primer 7 (Clarke & Gorley, 2015).

The Chao2 non‐parametric incidence‐based (FOO) richness estimator and 95% confidence intervals (Chao, 1987) were calculated with 1000 randomizations. To examine heterogeneity among samples, we compared the zero‐adjusted Bray–Curtis similarity index among individuals based on RRA. To assess dietary differences among spatial and temporal groups, comparisons of the similarity index were made among populations, sampling grids, years, and active seasons (spring = March–June; summer = July–August; fall = September–October). PPM have little to no above‐ground activity during the winter months. To make comparisons, we used analysis of similarities (ANOSIM: Clarke, 1993), which is a non‐parametric ranked analysis of a resemblance matrix with significance assessed with 9999 permutations. We examined which plant species contributed most to differences among significantly different groups using a similarity percentages routine (SIMPER; Clarke & Gorley, 2015), which decomposes an average Bray–Curtis similarity matrix into percentage contributions from each species. Resemblance data were also visualized with non‐metric multidimensional scaling with a minimum stress of 0.01.

To compare diet to seed availability observed in the plant cover and phenology surveys, we reduced diet genera to those that were observed at any time in survey plots in 2016 and 2017. The relative read abundance of each available plant in PPM diets was standardized as a proportion of the total reads in each trapping grid (individual fecal samples summed). Similarly, the proportion of each available plant (flowering, seeding, dead) in the habitat was standardized as a proportion of the total trapping grid. Because plant phenology data were collected only during the spring and summer, all fall diet data were removed from further analysis, resulting in 44 temporally and spatially matched diet and habitat samples. With these data, we subtracted the relative read abundance in diet from the proportion available on the trap plot to infer seed selection across seasons, years, and populations. We also calculated resemblance matrices (zero adjusted Bray–Curtis similarity) separately for diet and habitat availability and assessed the rank correlation between matrices using the RELATE procedure in Primer (Clarke & Gorley, 2015), which uses random permutations to test a hypothesis of no relation between multivariate patterns from two sets of samples. We further summarized diet and habitat with the Shannon diversity index (H′, Shannon & Weaver, 1949), which incorporates the number of species or taxa and their abundance in the sample, and Pielou's (1966) evenness index (J'), which represents a measure of proportional evenness among species or taxa in a sample. Paired *t*‐tests were used to test for differences in diet and habitat diversity indices.

## 3 RESULTS

We prepared and sequenced 222 PPM scat pellets and two mock samples. After filtering and removing one sample that failed, our final dataset contained 221 fecal samples (88 from EDSON and 133 from SSM; Table 1) with a per‐sample average of 22,545 reads assigned to reference sequences in the SDNHM BOLD database. Overall, we recovered 94 plant genera in the sequence database that were found with a frequency of at least 1% in a single fecal sample. After removing plant taxa with no recorded local observations, we retained 73 for further analysis (See Table A2 for the full list and overall relative read abundance and frequency of occurrence). Species‐accumulation curves plateaued between 50 and 100 fecal samples and reached a maximum of 75 diet taxa (SD 2.5). The number of diet taxa recovered in a single fecal sample averaged 3 (range 1–7) and did not differ between sites.

**TABLE 1:** Numbers of Pacific pocket mouse (PPM) fecal samples analyzed by population, sampling grid, year, and season.

Year/season	EDSON		SSM			Total
	Grid 663	Grid 819	Grid 1433	Grid 1450	Grid 1452	
**2016**						
Fall	6	6	–	–	–	12
Spring	15	10	10	9	11	55
Summer	12	7	6	12	3	40
**2017**						
Fall	7	10	9	1	4	31
Spring	3	1	16	13	12	45
Summer	2	9	9	10	8	38
**Total**	45	43	50	45	38	221

*Note*: There were no PPM trapped in Fall of 2016 at South San Mateo (SSM).

**FIGURE 2:**
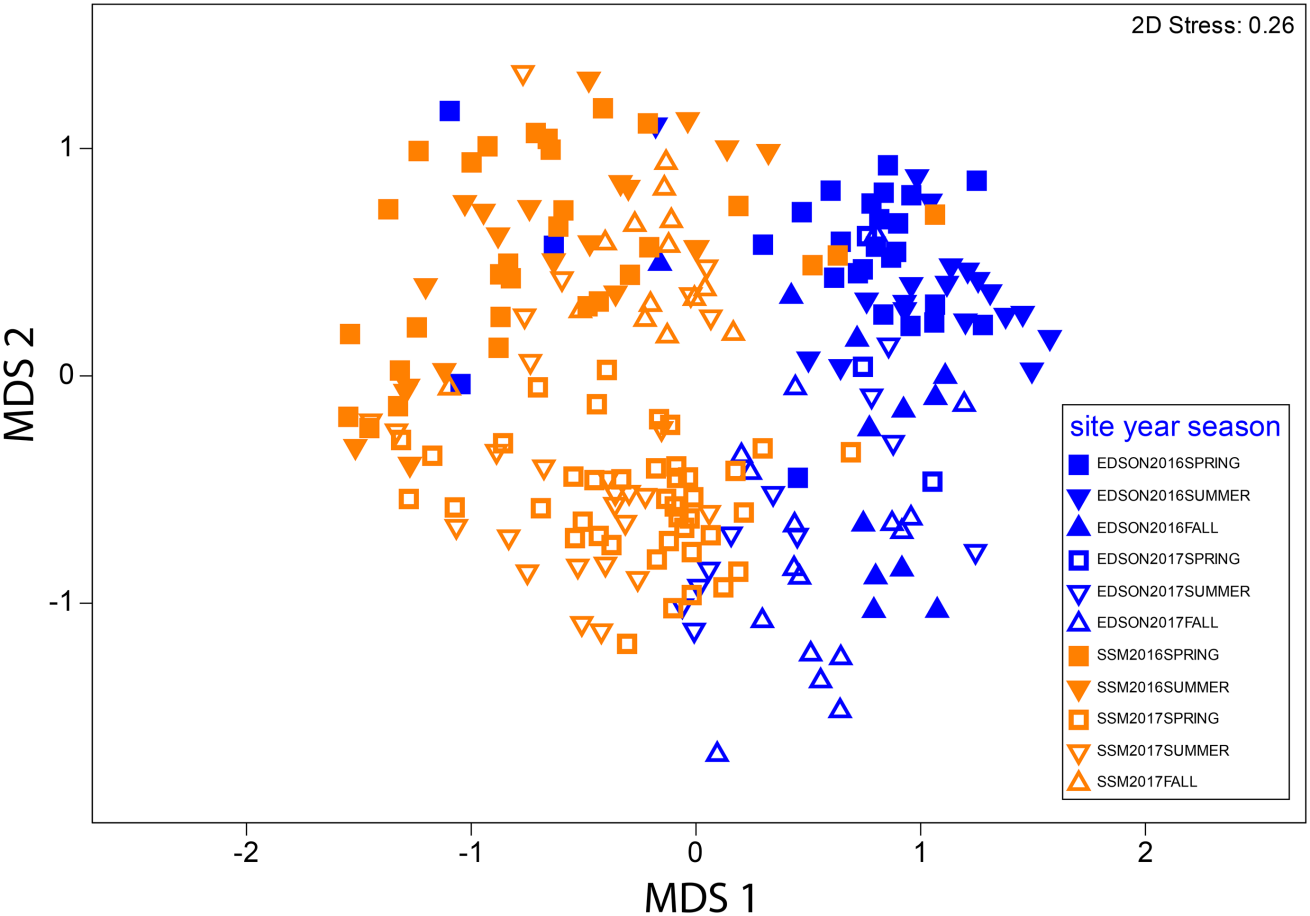
Non‐metric multi‐dimensional scaling plot of scat sample Bray–Curtis similarities. Samples are colored by site and symbolized by site, year, and season.

**FIGURE 3:**
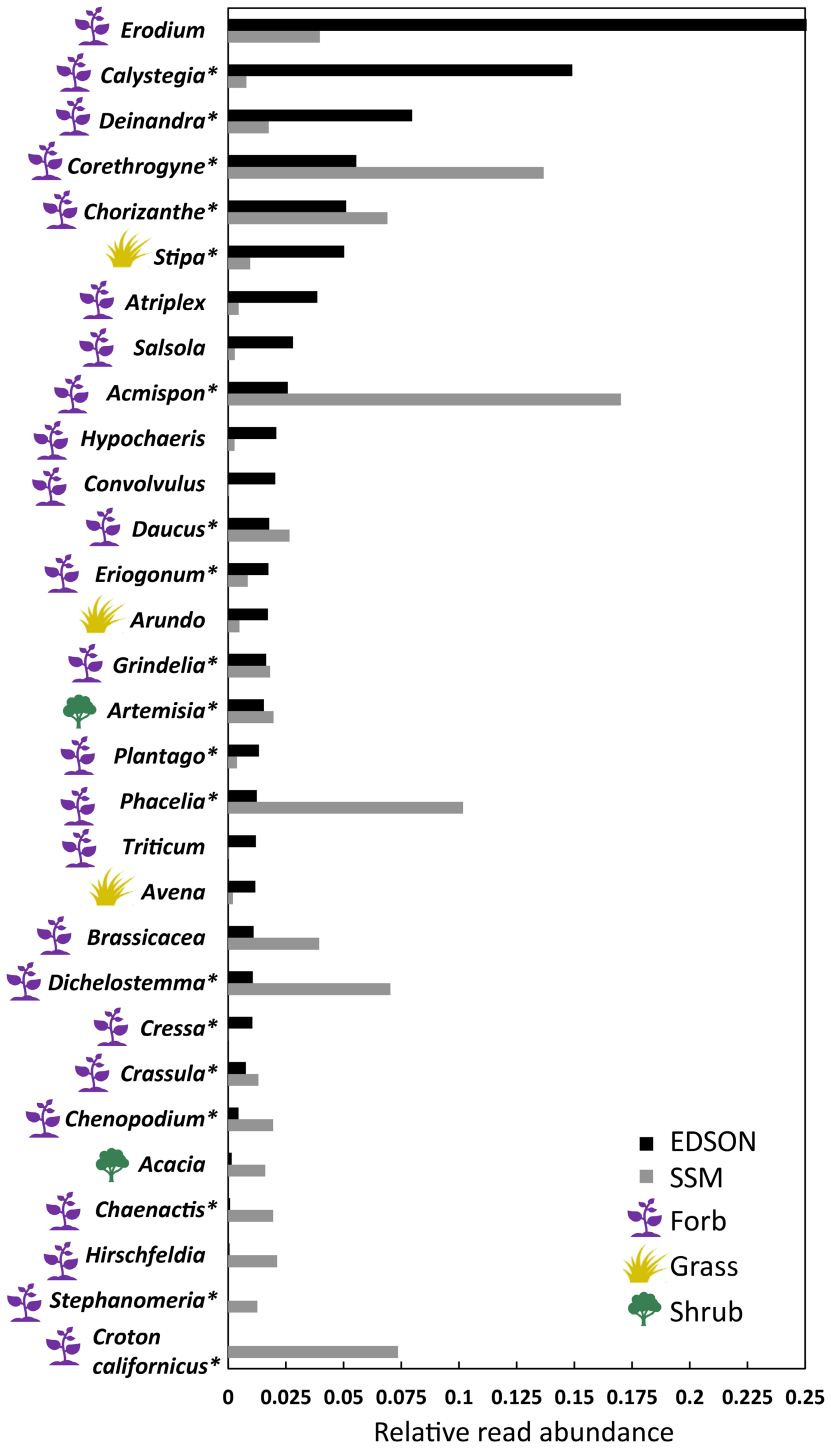
Relative read abundances of the top recovered plant genera (>1% total per site) were summed across all fecal samples at EDSON (black bars) and South San Mateo (SSM; gray bars). Plant growth types are indicated with colored icons (forb/perennial herb = purple, grass = gold, shrub/tree = green). Asterisks denote taxa that are native to southern California.

Forbs made up the largest RRA across sites, years, and seasons, ranging from about 70 to 90% of the total diet. Tests for differences in diet composition were statistically significant among sites (ANOSIM *R* = .379, *p* ≤ .001 from 9999 permutations; Figure 2), among grids within sites (EDSON *R* = .032, *p* ≤ .034; SSM *R* = .035, *p* ≤ .009), among years within sites (EDSON *R* = .385, *p* ≤ .001; SSM *R* = .347, *p* ≤ .001), and among seasons within sites (EDSON *R* = .252, *p* ≤ .001; SSM *R* = .062, *p* ≤ .006). Much of the difference between sites was explained by the higher prevalence of both *Erodium* and *Calystegia* in the diet in EDSON and the higher prevalence of *Acmispon*, *Corethrogyne*, and *Phacelia* in SSM (Table 2; Figure 3). *Croton californica* was also prevalent in diets in SSM. Its congener, *Croton setiger*, was detected in field surveys in EDSON but was not detected in diet. Annual and seasonal composition differences within populations can be found in Tables A2 and A3.

**TABLE 2:** Analysis of similarity (ANOSIM) statistical results and similarity percentages (SIMPER) across top taxa contributing to differences between sites EDSON and South San Mateo (SSM).

Populations						
ANOSIM: *R* = .379, *p* ≤ .001						
Average dissimilarity = 89.60						
	EDSON	SSM				
Taxon	Av.RRA	Av.RRA	Av.Diss	Diss.SD	Contrib%	Cum.%
*Erodium*	24.99	3.98	12.00	0.98	13.39	13.39
*Acmispon*	2.54	17.02	8.78	0.68	9.80	23.19
*Corethrogyne*	5.62	13.67	8.10	0.78	9.04	32.24
*Calystegia*	15.01	0.79	7.54	0.66	8.41	40.65
*Phacelia*	1.23	10.18	5.33	0.57	5.95	46.60
*Deinandra*	8.07	1.76	4.43	0.46	4.95	51.55
*Chorizanthe*	5.06	6.90	4.42	0.78	4.93	56.48
*Dichelostemma*	1.04	7.03	3.74	0.47	4.18	60.66
*Croton californicus*	0.00	7.36	3.68	0.55	4.11	64.76
*Stipa*	5.03	0.95	2.69	0.60	3.01	67.77
Brassicaceae	1.10	3.94	2.20	0.65	2.46	70.23

*Note*: For each plant taxon (Taxon), the average relative read abundance (Av. RRA) in each population, the average of the Bray–Curtis dissimilarities between all pairs of samples between sites (Av. Diss), the standard deviation of dissimilarities (Diss.SD), the percent contribution (Contrib%), and the cumulative percent (Cum.%) are reported.

**FIGURE 4:**
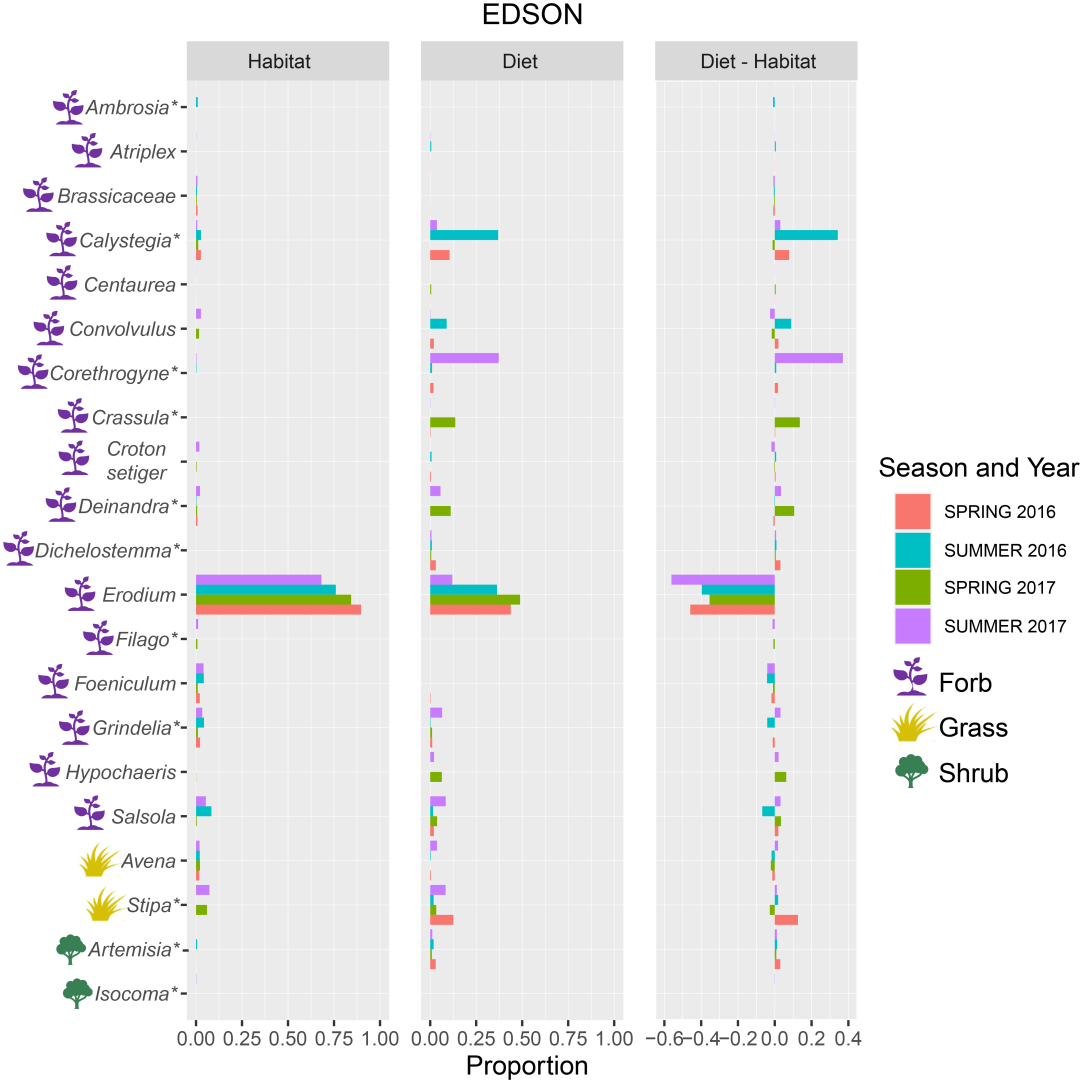
Proportions of plant genera available in habitat, recovered in PPM diet, and the differences between diet and habitat in EDSON during the spring and summer activity periods of 2016 and 2017. Plant growth types are indicated with colored icons (forb/perennial herb = purple, grass = gold, shrub/tree = green). Asterisks denote taxa that are native to southern California.

**FIGURE 5:**
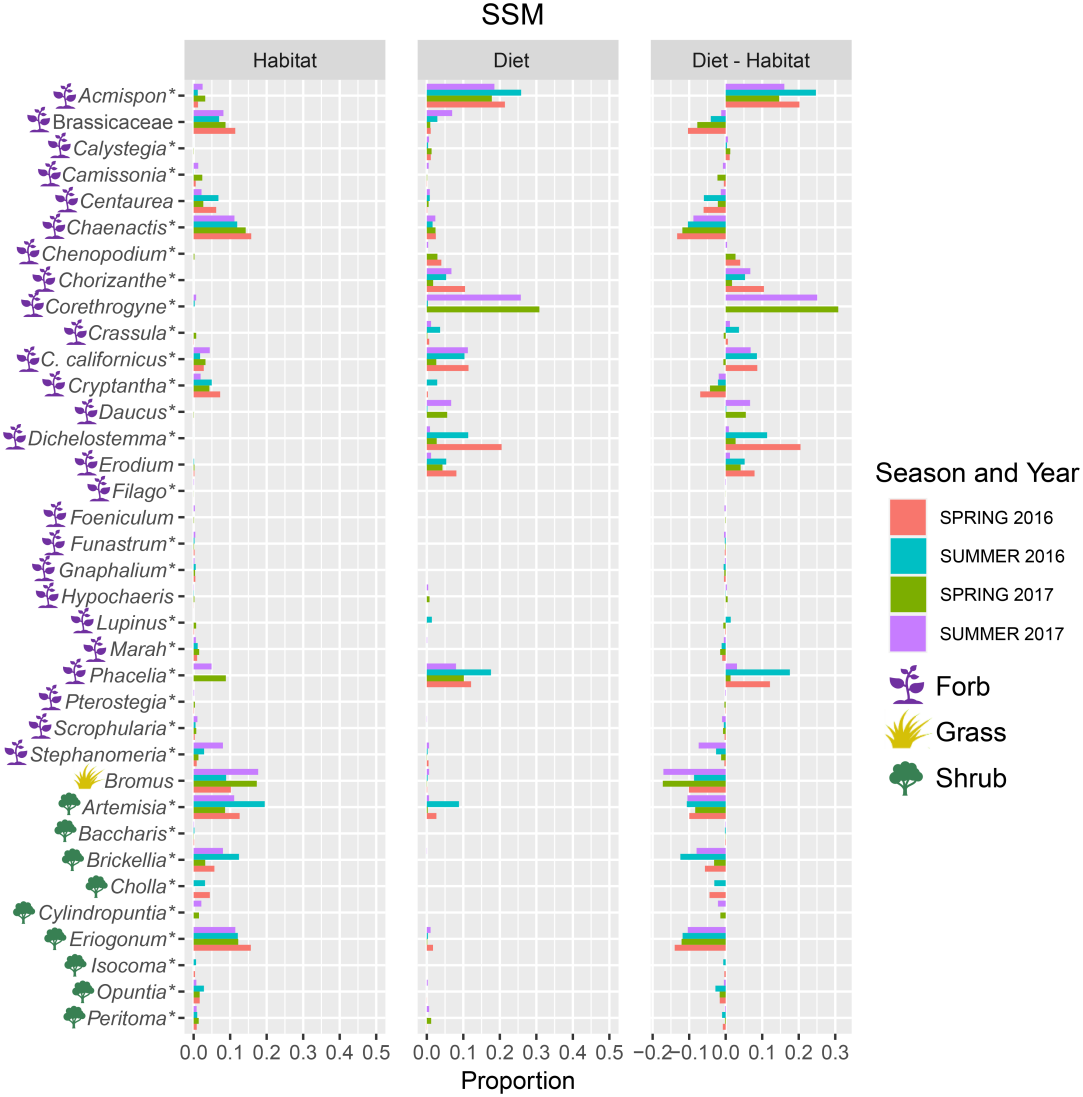
Proportions of plant genera available in habitat, recovered in PPM diet, and the difference between diet and habitat in South San Mateo (SSM) during the spring and summer activity periods of 2016 and 2017. Plant growth types are indicated with colored icons (forb/perennial herb = purple, grass = gold, shrub/tree = green). *C. californicus* = *Croton californicus*. Asterisks denote taxa that are native to southern California.

Forty‐four of the 73 plant taxa recovered in fecal samples were identified and counted in the trap‐centered survey sample grids: 20 in EDSON and 34 in SSM. Variation across habitat availability and diet proportions was significantly correlated overall (Rho = .476, *p* ≤ .001) and within each site (EDSON: Rho = .442, *p* ≤ .013; SSM: Rho = .104, *p* ≤ .047). In Edson, *Erodium* was found in the highest proportions in both diet and habitat (Figure 4). Across seasons and years in EDSON, *Artemisia*, *Atriplex*, *Convolvulus*, *Corethrogyne*, and *Hypochaeris* were highly overrepresented in diet compared to habitat availability (>10×), while Brassicaceae and *Foeniculum* were underrepresented (Figure 4). In SSM, the perennial herb *Acmispon* comprised the largest proportion of diet but a relatively small proportion of habitat availability (Figure 5). In addition to *Acmispon*, the forbs *Calystegia*, *Chenopodium*, *Corethrogyne*, *Daucus*, and *Erodium* were overrepresented in diet compared to their habitat availability, while the non‐native grass *Bromus*, the shrubs *Brickelia*, *Eriogonum*, *Opuntia* cactus, and the wild cucumber vine *Marah* were highly underrepresented in diet (Figure 5). In EDSON, both the Shannon diversity index (H′) and evenness (J') were higher in diet than habitat availability (H′: Diet $\bar{x}$ = 1.57, Habitat $\bar{x}$ = 0.82, *t* = 5.88, 2‐sided *p* ≤ .0001, 14 df; J': Diet $\bar{x}$ = 0.67, Habitat $\bar{x}$ = 0.37, *t* = 6.89, *p* ≤ .0001). Both metrics were lower in diet than habitat availability in SSM (H′: Diet $\bar{x}$ = 1.86, Habitat $\bar{x}$ = 2.15, *t* = −3.65, 2‐sided *p* ≤ .001, 28 df; J': Diet $\bar{x}$ = 0.71, Habitat $\bar{x}$ = 0.77, *t* = −2.17, *p* ≤ .038).

## 4 DISCUSSION

Overall, the diet of PPM appeared to be broad, with a wide representation of plant genera and high diversity among samples, mirroring previous metabarcoding results (Iwanowicz et al., 2016) and confirming a wider breadth than fecal microscopy (Meserve, 1976). Detected differences among seasons, years, and sites are not surprising, as annual plant abundance and distributions (and thus seed availability) can vary dramatically over time and space in southern California coastal sage scrub and chaparral communities, particularly in response to disturbance by fire, drought, and nitrogen deposition (Jacobsen & Pratt, 2018; McEachern et al., 2007; Zedler et al., 1983).

### 4.1 Diet reflects major differences in plant communities over space and time

Overall, observed differences in diet between sites appear to largely reflect major differences in plant communities. These differences may be due to different localized disturbance regimes. For example, EDSON has been subject to more frequent and recent fires and other disturbances and thus has fewer shrubs and higher proportions of forbs and open ground than SSM (Brehme et al., 2018). Yearly and seasonal differences in diet composition were more pronounced in EDSON than in SSM, possibly reflecting the differences in forb availability in a drought (2016) versus wet year (2017), as well as post‐disturbance turnover at this site. In addition, PPM diet and availability were more strongly correlated in EDSON than in SSM, a pattern that may be largely driven by the high proportion of *Erodium* in both the habitat and diet at EDSON. Habitat in SSM was generally characterized by more mature and species‐rich coastal sage scrub vegetation and a larger proportion of perennial shrub cover, which appeared to be largely avoided in the PPM diet. Although still statistically significant, the amount of variation explained by years and seasons was lower here than in EDSON (generally lower *R*‐values), possibly reflecting more stable seed resources.

### 4.2 Diet preference

This is the first study of PPM to pair plant phenology surveys with diet metabarcoding to estimate resource selection. Significant similarity in diet and habitat availability at survey grids suggests that, to a large extent, PPM diet reflects local seed availability. However, we still detected some differences in above‐ground seed availability and proportion in fecal samples that indicate diet preferences for some forbs, perennial herbs, and native bunch grasses over perennial shrubs and non‐native grasses. These results, along with the complimentary results of a concurrent occupancy study (Brehme et al., In Review), described in more detail below, can help us to better understand relationships between food resources and the population dynamics of PPM. Our results also provide information about specific forbs and perennial herbs that can be incorporated into habitat restoration and site selection and preparation efforts for establishing new populations (Chock et al., 2022).

Higher forb cover is a primary predictor of increased probabilities of local PPM occupancy and colonization (Brehme et al., 2018, 2020). Correspondingly, forbs dominated the diet of PPM across sites and years, and almost all forbs documented in the habitat were detected in the PPM scat. As heteromyids, PPM cache or hoard seeds, which may increase the temporal availability of these limited resources (Price et al., 2000). Forb seeds are typically high in moisture, lipids, and protein, which are seed traits preferred by scatter‐hoarding rodents (Reichman & Price, 1993; Ríos et al., 2012; Wang & Chen, 2012). Preferred forb and perennial herb seeds (those that were reflected in the PPM diet in higher proportions than in the habitat) in one or more seasons and sites may be considered important food sources for PPM and could be included in seed palettes for restoration. These include aster (*Corethrogyne*), wild hyacinth (*Dichelostemma*), California croton (*C. californicus*), deerweed (*Acmispon*), morning glory (*Calystegia macrostegia*), storksbill filaree (*Erodium*), spineflowers (*Chorizanthe*), pigmyweed (*Crassula*), tarweed (*Deinandra*), cat's ear (*Hypochaeris*), wild carrot (*Daucus*), and scorpionweed (*Phacelia*).

Conversely, higher non‐native grass cover has been a primary predictor of decreased probability of PPM occupancy and increased probability of localized extinction across sites (Brehme et al., In Review). Large contiguous stands of annual grasses impede the growth of other plant species (Flory & Clay, 2010) and may hinder PPM movement and foraging success. Although other pocket mouse species may forage on *Bromus* seed (e.g., Richardson et al., 2013), our study suggests that non‐native grasses such as *Bromus* are not preferred in the PPM diet. Further, studies of other species have found that Brome grasses can have little nutritional value in comparison to forbs (e.g., Drake et al., 2016 [tortoise]; Hall, 2012 [deer mouse]). We did find evidence of native bunch grass (*Stipa*) in fecal samples, although in low proportions compared to forbs. Other heteromyids have been shown to prefer native grass to invasive grass seeds, which can lead to reduced recruitment of native grasses and continued establishment of non‐native grasses (Lucero et al., 2015; Lucero & Callaway, 2018). Strong negative associations with occupancy combined with low diet preference support non‐native grass removal and control in PPM habitats. Finally, shrub cover has not typically been correlated with PPM occupancy, and our study results show that seeds of shrubs were largely avoided in the PPM diet, including wild buckwheat (*Eriogonum*), brickelbush (*Brickellia*), and cacti (*Opuntia* and *Cholla*). Although PPM did not consume a high proportion of shrub and non‐native grass seeds during our 2016–17 sample period, it is possible that these seeds may be used when other preferred seed resources are low (Meserve, 1976).

### 4.3 Diet breadth

Almost half of the total plant taxa detected in the PPM diet were not surveyed in plots during the study period. However, these missing taxa had very small RRAs, and the most prevalent diet items (>2% RRA) were recorded in field surveys. This could suggest that PPM have a wider foraging range than study plot size or that a more diverse seed bank is accessible to PPM than represented in the above‐ground plant biomass at a given time. Soil seed banks can be more diverse than the above‐ground vegetation types in coastal sage scrub (Cox & Allen, 2007) and are typically persistent over time (Parker & Kelly, 1989). A diverse available seed bank, along with seed caching behaviors, may contribute to the high richness we recovered in our diet. Additional soil seed bank analysis and seed capture studies could be ideal for determining differences in above‐ground versus soil seed banks. However, we did not sample PPM habitat in this manner due to the high risks of disturbing habitat and food resources and negatively impacting this critically endangered species. Additionally, locally rare taxa could have been overlooked in field surveys or misidentified by genetic methods. Although the ITS2 region has been shown to have a high success rate (97%) in identifying plant genera (Yao et al., 2010), we cannot rule out occasional errors in available sequence databases (e.g., Meiklejohn et al., 2019). Future work could target an additional barcoding gene, such as the trnL P6 loop, as some studies have used multiple regions to improve identification (De Barba et al., 2014; Lopes et al., 2015).

### 4.4 Links between food limitations and population declines

The impacts of diet shifts on individual nutrition and survivorship in wild populations of PPM are unknown. To assist in managing existing wild populations, it could be beneficial to identify links between habitat characteristics, plant phenology, PPM diet composition, and patterns of population growth or decline. Often, these are bottom‐up processes that can impact the fitness of organisms by altering the availability of resources (White, 2008). In 2018, we observed a link between climate, forb phenology, and PPM reproductive phenology when southern California experienced a severe and early drought year with only 5.9 in of rainfall (Brehme et al., 2020). Our plant phenology surveys documented an early forb die‐off that was associated with early extreme drought conditions. Many forbs died before producing seed. In contrast to the previous 6 years of monitoring, few PPM adults showed any signs of reproductive activity (estrus/perforate females, scrotal males), and no juveniles or subadults were captured throughout the season. The seed resources may have been too limited to support the high energy and nutrient demands of reproduction. The following year, both sites experienced a substantial population decline (40–51%, Brehme et al., 2021).

Climate predictions for southern California include increases in mean temperature and heat wave frequency, with more intensity and longer duration, more extreme weather and flooding events, and increased frequency and size of wildfires (e.g., Cayan et al., 2010). The changes to the timing of rainfall and its effect upon spring forb growth could result in more variability in the availability of seed resources required for reproduction. Therefore, perennial herbs and shrubs may be important seed resources during these times. Further research on diet as it relates to PPM demography and phenology over time could help to determine dietary changes during extreme changes in resources. However, the years of our study did include a drought (2016) and an extremely wet (2017) year. In both years and sites, PPM preferred a diverse diet dominated by forbs. Together, these trends suggest that open and diverse forb and native grassland habitat are important for both PPM food resources and occupancy. Management and restoration to optimize these habitat conditions could assist in recovery efforts for this endangered species.

## AUTHOR CONTRIBUTIONS

**Amy G. Vandergast:** Conceptualization (equal); data curation (supporting); formal analysis (equal); funding acquisition (equal); methodology (supporting); supervision (equal); visualization (equal); writing – original draft (lead); writing – review and editing (lead). **Cheryl S. Brehme:** Conceptualization (equal); formal analysis (equal); funding acquisition (equal); investigation (equal); methodology (equal); visualization (equal); writing – review and editing (equal). **Deborah Iwanowicz:** Data curation (lead); investigation (equal); methodology (lead); supervision (equal); writing – original draft (supporting); writing – review and editing (equal). **Robert S. Cornman:** Formal analysis (equal); methodology (equal); visualization (supporting); writing – original draft (supporting); writing – review and editing (equal). **Devin Adsit‐Morris:** Formal analysis (supporting); investigation (supporting); methodology (equal); writing – review and editing (equal). **Robert N. Fisher:** Conceptualization (supporting); funding acquisition (equal); supervision (equal); writing – review and editing (equal).

## CONFLICT OF INTEREST STATEMENT

The authors declare that there are no competing interests.

## Data Availability

Machine‐processed sequencing output and sample data are available from NCBI under BioProject PRJNA801232.

## APPENDIX A

**TABLE A1:** Full list of plant taxa retained and their relative read abundance and frequency of occurrence across all fecal samples.

Plant taxon	Relative read abundance	Frequency of occurrence
*Erodium*	12.47	67.57
*Acmispon*	11.23	52.25
*Corethrogyne*	10.42	47.75
*Phacelia*	6.59	36.04
*Calystegia*	6.45	40.54
*Chorizanthe*	6.18	57.21
*Dichelostemma*	4.64	36.49
*Croton californicus*	4.41	31.53
*Deinandra*	4.25	37.84
*Brassicaceae*	2.80	38.29
*Stipa*	2.58	32.88
*Daucus*	2.31	34.68
*Atriplex*	1.82	12.61
*Artemisia*	1.80	36.04
*Grindelia*	1.75	34.68
*Chenopodium*	1.35	13.06
*Salsola*	1.30	22.97
*Hirschfeldia*	1.29	18.47
*Eriogonum*	1.21	18.92
*Chaenactis*	1.20	18.47
*Crassula*	1.09	14.86
*Acacia*	1.03	26.13
*Hypochaeris*	1.00	18.47
*Arundo*	0.99	21.62
*Convolvulus*	0.84	5.41
*Stephanomeria*	0.76	9.91
*Plantago*	0.76	17.12
*Avena*	0.60	14.41
*Capsella*	0.57	11.71
*Phalaris*	0.57	13.06
*Triticum*	0.49	6.31
*Cressa*	0.43	4.95
*Cirsium*	0.32	14.41
*Polycarpon*	0.30	4.50
*Centaurea*	0.30	9.91
*Eucalyptus*	0.30	1.35
*Cucurbita*	0.29	5.41
*Cryptantha*	0.29	4.05
*Peritoma*	0.28	2.70
*Bromus*	0.24	6.31
*Ageratina*	0.23	7.66
*Amsinckia*	0.22	3.15
*Rosa*	0.17	3.15
*Cenchrus*	0.16	1.80
*Festuca*	0.16	5.41
*Lupinus*	0.12	0.90
*Croton setiger*	0.12	2.70
*Ambrosia*	0.11	1.80
*Nuttallanthus*	0.11	1.80
*Helianthus*	0.11	4.05
*Rafinesquia*	0.10	2.70
*Gilia/Eriastrum*	0.09	0.45
*Encelia*	0.08	0.45
*Raphanus*	0.08	3.60
*Camissonia*	0.08	1.35
*Setaria*	0.08	1.35
*Lactuca*	0.05	1.35
*Taraxacum*	0.05	3.60
*Kochia*	0.04	1.80
*Marah*	0.04	2.25
*Foeniculum*	0.04	0.90
*Opuntia*	0.03	0.90
*Euphorbia*	0.03	0.90
*Brickellia*	0.03	1.35
*Scrophularia*	0.03	1.80
*Sambucus*	0.03	1.80
*Malosma*	0.02	1.35
*Quercus*	0.02	1.35
*Rhus*	0.02	1.35
*Baccharis*	0.01	0.90
*Carthamus*	0.01	0.45
*Salix*	0.01	0.90
*Schismus*	0.01	0.45

*Note*: In a few cases a recovered taxon had an alternate recognized name (Calflora Database https://www.calflora.org/; accessed 27 May 2021) In these cases both recognized names are listed here.

**TABLE A2:** EDSON Analysis of similarity (ANOSIM) statistical results and similarity percentages (SIMPER) across top taxa contributing to differences between years and seasons.

EDSON years						
ANOSIM: *R* = .390, *p* ≤ .001						
Average dissimilarity = 85.26						
	2016	2017				
Taxon	Av.RRA	Av.RRA	Av.Diss	Diss/SD	Contrib%	Cum.%
*Erodium*	31.51	13.88	14.72	1.23	17.27	17.27
*Calystegia*	21.09	3.90	10.91	0.88	12.79	30.06
*Deinandra*	0.27	21.69	10.77	0.77	12.63	42.69
*Corethrogyne*	1.70	12.43	6.52	0.57	7.65	50.34
*Stipa*	6.10	3.11	3.74	0.75	4.38	54.72
*Chorizanthe*	5.42	4.57	3.64	0.78	4.27	58.99
*Atriplex*	5.77	0.45	3.06	0.34	3.59	62.58
*Hypochaeris*	0.00	5.81	2.90	0.72	3.41	65.98
*Salsola*	1.90	4.44	2.65	0.73	3.11	69.10
*Acmispon*	3.01	1.81	2.16	0.42	2.54	71.63

EDSON Seasons						
ANOSIM: *R* = 0.257, *p* ≤ .001						
Groups FALL & SPRING						
Average dissimilarity = 86.21						
	FALL	SPRING				
Taxon	Av.RRA	Av.RRA	Av.Diss	Diss/SD	Contrib%	Cum.%
*Erodium*	8.70	40.57	18.10	1.43	21.00	21.00
*Deinandra*	21.16	1.47	10.71	0.74	12.42	33.42
*Calystegia*	12.70	8.46	8.69	0.71	10.08	43.50
*Atriplex*	11.44	0.04	5.73	0.48	6.64	50.14
*Stipa*	0.75	10.18	4.99	0.86	5.78	55.93
*Chorizanthe*	5.52	6.95	4.28	0.86	4.97	60.90
*Acmispon*	1.97	4.93	3.07	0.46	3.57	64.46
*Hypochaeris*	4.90	0.81	2.61	0.63	3.03	67.49
*Daucus*	3.82	0.35	1.96	0.76	2.27	69.76
*Salsola*	2.41	2.15	1.94	0.74	2.25	72.01

Groups FALL & SUMMER						
Average dissimilarity = 83.68						
	FALL	SUMMER				
Taxon	Av.Abund	Av.Abund	Av.Diss	Diss/SD	Contrib%	Cum.%
*Calystegia*	12.70	23.49	13.10	1.07	15.65	15.65
*Erodium*	8.70	25.70	11.91	1.11	14.23	29.89
*Deinandra*	21.16	1.73	10.49	0.73	12.53	42.42
*Corethrogyne*	1.90	13.19	6.84	0.60	8.17	50.59
*Atriplex*	11.44	0.36	5.80	0.49	6.94	57.53
*Chorizanthe*	5.52	2.87	3.20	0.79	3.82	61.35
*Salsola*	2.41	3.86	2.54	0.71	3.04	64.39
*Hypochaeris*	4.90	0.65	2.52	0.61	3.01	67.40
*Convolvulus*	0.00	4.69	2.34	0.40	2.80	70.20

Groups SUMMER & SPRING						
Average dissimilarity = 73.13						
	SUMMER	SPRING				
Taxon	Av.Abund	Av.Abund	Av.Diss	Diss/SD	Contrib%	Cum.%
*Erodium*	25.70	40.57	15.23	1.35	20.83	20.83
*Calystegia*	23.49	8.46	12.32	1.04	16.85	37.68
*Corethrogyne*	13.19	1.46	6.98	0.59	9.54	47.22
*Stipa*	4.00	10.18	5.31	0.96	7.27	54.48
*Chorizanthe*	2.87	6.95	3.94	0.73	5.39	59.88
*Convolvulus*	4.69	1.38	2.84	0.47	3.89	63.76
*Acmispon*	0.83	4.93	2.65	0.41	3.62	67.38
*Salsola*	3.86	2.15	2.60	0.64	3.56	70.94

*Note*: For each plant taxon (Taxon), the average relative read abundance (Av. RRA) in each population, the average of the Bray–Curtis dissimilarities between all pairs of samples between sites (Av. Diss), the standard deviation of dissimilarities (Diss.SD), the percent contribution (Contrib%), and the cumulative percent (Cum.%) are reported.

**TABLE A3:** South San Mateo (SSM) analysis of similarity (ANOSIM) statistical results and similarity percentages (SIMPER) across top taxa contributing to differences between years and seasons.

SSM years						
ANOSIM *R* = .347, *p* ≤ .001						
Average dissimilarity = 84.82						
	2016	2017				
Taxon	Av.RRA	Av.RRA	Av.Diss	Diss/SD	Contrib%	Cum.%
*Acmispon*	20.05	15.13	12.57	0.91	14.82	14.82
*Corethrogyne*	0.11	22.11	11.03	1.06	13.00	27.83
*Phacelia*	13.12	8.34	8.40	0.78	9.90	37.72
*Dichelostemma*	15.64	1.68	7.84	0.70	9.25	46.97
*Croton californicus*	9.86	5.81	6.03	0.80	7.11	54.08
*Chorizanthe*	7.62	6.46	5.16	0.82	6.09	60.17
*Erodium*	5.90	2.78	3.57	0.57	4.21	64.38
*Brassicaceae*	2.16	5.05	2.87	0.80	3.39	67.77
*Artemisia*	4.16	0.60	2.14	0.56	2.52	70.28

SSM Seasons						
ANOSIM: *R* = .062, *p* ≤ .006						
Groups SPRING & FALL						
Average dissimilarity = 83.95						
	SPRING	FALL				
Taxon	Av.RRA	Av.RRA	Av.Diss	Diss/SD	Contrib%	Cum.%
*Acmispon*	17.39	9.26	10.52	0.77	12.53	12.53
*Chorizanthe*	5.00	21.90	9.78	1.44	11.65	24.18
*Corethrogyne*	16.38	0.98	8.12	0.81	9.68	33.86
*Phacelia*	10.52	5.64	6.71	0.72	7.99	41.85
*Croton californicus*	5.92	8.53	5.53	0.75	6.59	48.44
*Dichelostemma*	9.72	0.74	4.84	0.52	5.77	54.21
*Stephanomeria*	0.23	9.40	4.70	0.87	5.60	59.81
*Brassicaceae*	1.53	10.36	4.67	1.61	5.56	65.37
*Hirschfeldia*	0.56	7.54	3.64	1.48	4.33	69.70
*Erodium*	5.29	2.83	2.82	0.53	3.35	73.05

Groups SUMMER & FALL						
Average dissimilarity = 80.30						
	SUMMER	FALL				
Taxon	Av.RRA	Av.RRA	Av.Diss	Diss/SD	Contrib%	Cum.%
*Acmispon*	18.73	9.26	10.89	0.80	13.56	13.56
*Chorizanthe*	5.33	21.90	9.29	1.38	11.56	25.12
*Phacelia*	10.99	5.64	6.96	0.67	8.66	33.79
*Croton californicus*	9.14	8.53	6.73	0.77	8.38	42.17
*Corethrogyne*	13.37	0.98	6.70	0.72	8.34	50.51
*Brassicaceae*	5.64	10.36	4.87	1.55	6.07	56.58
*Stephanomeria*	0.41	9.40	4.66	0.88	5.81	62.39
*Hirschfeldia*	2.85	7.54	3.78	1.41	4.70	67.09
*Dichelostemma*	4.89	0.74	2.59	0.40	3.22	70.31

Groups SUMMER & SPRING						
Average dissimilarity = 79.49						
	SUMMER	SPRING				
Taxon	Av.RRA	Av.RRA	Av.Diss	Diss/SD	Contrib%	Cum.%
*Acmispon*	18.73	17.39	12.70	0.91	15.97	15.97
*Corethrogyne*	13.37	16.38	10.25	1.04	12.90	28.87
*Phacelia*	10.99	10.52	8.31	0.75	10.45	39.32
*Dichelostemma*	4.89	9.72	6.19	0.62	7.78	47.11
*Croton californicus*	9.14	5.92	5.81	0.74	7.30	54.41
*Chorizanthe*	5.33	5.00	3.93	0.73	4.95	59.36
*Erodium*	2.37	5.29	3.14	0.55	3.95	63.31
*Brassicaceae*	5.64	1.53	3.01	0.74	3.79	67.09
*Daucus*	3.07	2.88	2.40	0.68	3.02	70.11

*Note*: For each plant taxon (Taxon), the average relative read abundance (Av. RRA) in each population, the average of the Bray–Curtis dissimilarities between all pairs of samples between sites (Av. Diss), the standard deviation of dissimilarities (Diss.SD), the percent contribution (Contrib%), and the cumulative percent (Cum.%) are reported.
